# Myoepithelioma of the Soft Palate: A Case Report

**DOI:** 10.1155/2013/642806

**Published:** 2013-10-02

**Authors:** Ajay Kumar Yadav, Jeyaseelan Nadarajah, S. H. Chandrashekhara, Vishal Dnyandeo Tambade, Sudeep Acharya

**Affiliations:** Department of Radiodiagnosis, All India Institute of Medical Sciences, New Delhi 110029, India

## Abstract

Myoepitheliomas are rare benign tumors of myoepithelial cell origin, most commonly seen in parotid gland. These tumors are also reported in oral cavity, soft palate being the most common site of involvement. Imaging findings are nonspecific, and histopathology is necessary to differentiate from other tumors. Our case showed mildly enhancing well-circumscribed mass in soft palate with histological findings consistent with myoepithelioma. The aim of this case report is to increase the awareness about this rare benign tumor regarding its morphological, histopathological, and radiological features along with its possible differential diagnosis.

## 1. Introduction

Myoepitheliomas are rare benign neoplasm composed of ectodermally derived contractile smooth muscle cells, that is, myoepithelial cells which lack ductal differentiation. Myoepithelial cells are present in salivary glands, breast, larynx, and sweat glands of skin; hence, myoepitheliomas are reported in these sites [1–3]. About 50% of salivary gland myoepitheliomas arise in parotid gland followed by sublingual gland (33%) and submandibular gland (13%) [4]. Myoepitheliomas arising in the oral cavity are very rare constituting 1.5% of all salivary gland tumors [5]. Myoepithelioma occurring in minor salivary glands of oral cavity accounts for 26% of all salivary gland myoepitheliomas and palate being the most common site [6–9]. The tumors mostly present as asymptomatic, slowly progressive masses over a period of months to years in the patient with average age in the fourth decade [1]. Myoepitheliomas were considered as variant of pleomorphic adenoma; however, it is considered as separate clinical entity according to WHO since 1991 [10]. In myoepitheliomas, the ducts constitute less than 5% of the section. Chondromyxoid matrix or osteoid formation is not seen in myoepitheliomas, however it is characteristically present in pleomorphic adenoma. Here, we present a very rare case of myoepithelioma arising from soft palate along with their imaging features, histological findings, and management.

## 2. Case Presentation

**Figure 1:**
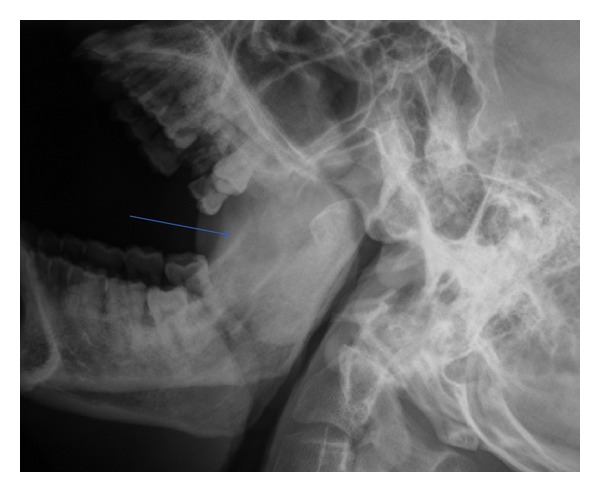
Plain radiograph of soft tissue of nasopharynx lateral view showing well defined round mass (marked by thin arrow) in the oropharyngeal region with no calcification or cavitation and causing significant narrowing of oropharynx.

**Figure 2:**
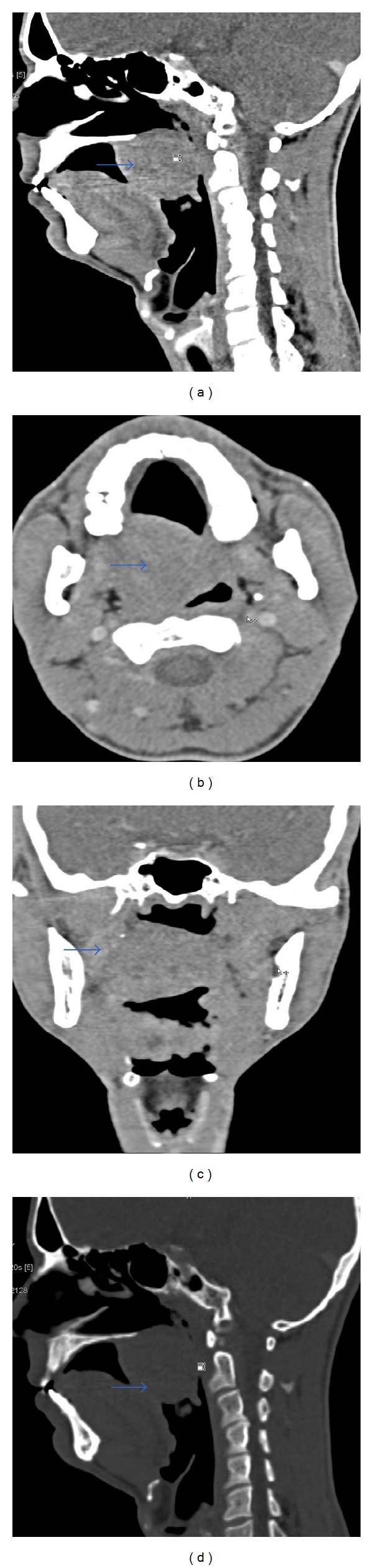
Contrast enhanced CT soft tissue window (a) sagittal section, (b) axial section, (c) coronal section, and (d) bone window showing well defined mildly enhancing heterogeneous soft tissue mass (marked by arrow) arising from soft palate reaching up to the posterior pharyngeal wall causing significant narrowing of nasopharyngeal and oropharyngeal airway. No cystic area or fat or calcification within the mass. The mass is abutting the hard plate with no erosion or destruction.

A 40-year-old nonalcoholic, nonsmoker male presented with an asymptomatic slowly progressive palatal mass for four years. There was no history dysphagia, odynophagia, sleep apnea, voice change, weight loss, loss of appetite, and fever. The clinical examination revealed a firm, nontender, nonpulsatile, round, pinkish mass originating from the midline and right side of soft palate with no ulceration or erosion of overlying mucosa. No significant cervical lymphadenopathy was noted. X-ray soft tissue of nasopharynx lateral view (Figure 1) showed well defined mass in oropharyngeal region closely abutting the posterior pharyngeal wall and causing significant oropharyngeal airway compromise. Contrast enhanced CT (64 slice MDCT) was performed after intravenous administration of 70 mL of iodinated nonionic contrast into antecubital vein. The CT scan (Figure 2) showed well-defined heterogeneous mildly enhancing soft tissue mass arising from soft palate (measuring 5.4 × 4.4 × 4.2 cm) causing significant nasopharyngeal and oropharyngeal airway compromise abutting the hard palate with no obvious involvement of hard palate. There is no calcification, cystic component, or fat within the mass. The mass is closely abutting the base of the tongue and posterior pharyngeal wall with maintained fat plane between them. No significant cervical lymphadenopathy noted. Based on CT findings, diagnosis of benign palatal mass was made. The patient underwent incisional biopsy of the palatal mass. The histological examination revealed tumors arising from minor salivary gland with predominant population of monomorphic plasmacytoid as well as spindle cell forming areas of loose clusters. Scant basement membrane-like material was identified with no epithelial cells, cellular pleomorphism, cellular atypia, or mitotic figures. The tumor was positive for vimentin, smooth muscle antigen, and S-100 on immunohistological staining. The findings were compatible with myoepithelial tumor (myoepithelioma). The patient underwent successful surgery for this soft palate tumor and histological diagnosis was reconfirmed.

## 3. Discussion

 Myoepitheliomas show four different morphological patterns which include nonmyxoid (solid), myxoid (pleomorphic adenoma like), reticular (canalicular like), and mixed [11]. The cellular patterns of myoepitheliomas consists of plasmacytoid cells, spindle cells, epitheloid cells, and clear cell patterns which do not account for differences in recurrence rate, biological behavior, or the patient age. In oral cavity, plasmacytoid cell type is more commonly seen while spindle cell type is more frequently seen in parotid gland [8]. Myoepithelial cells are most commonly seen in salivary glands. It is also seen in extrasalivary gland tissues like breast, skin, lung, and larynx. Myoepitheliomas occurring in both salivary and extrasalivary tissues showed similar morphological and immunohistological characteristics. 

Myoepithelioma should be differentiated from its malignant counterpart that is, malignant myoepithelioma which is more aggressive and show recurrence even after adequate treatment. Histopathologically presence of cellular atypia, cellular pleomorphism, cellular necrosis, increased mitotic figures, invasive growth pattern, or combination of these favour the diagnosis of malignant myoepithelioma [12]. 

Myoepitheliomas of soft palate needs to be differentiated from other tumors of soft palate like pleomorphic adenoma, neurinomas, hemangiomas, malignant tumors, metastatic tumors, lymphoma, solitary fibrous tumor, nerve sheath tumors, fibrous histiocytoma, paraganglioma, leiomyoma, leiomyosarcoma, hemangiopericytoma, and other inflammatory diseases [12, 13]. Many of these lesions share common clinical and radiological features, so biopsy is needed for confirmation of diagnosis of myoepithelioma, as it is difficult to differentiate myoepithelioma from other salivary gland tumors such as pleomorphic adenoma [7].

Myoepitheliomas showed varying enhancement pattern on CT: faint enhancement, no significant enhancement or marked enhancement. Factors influencing enhancement pattern of myoepithelioma include histological component, stroma, vascularity, and histological cell type. The cellular myoepithelioma with fibrous stroma being more vascular showed more enhancement than those myoepithelioma being rich in myxoid stromal component. In our case the tumor showed mild heterogeneous enhancement after contrast administration. Enhancement patterns may have a role in differentiating the slow growing well demarcated masses of soft palate [7, 14, 15].

Pleomorphic adenoma is the most common minor salivary gland constituting 40% of total cases having epithelial and ductal cells in its tissue. Presence of chondromyxoid matrix is considered most specific for pleomorphic adenoma while it is absent in myoepithelioma along with absence of glanduloductal differentiation [16]. Peripheral nerve sheath tumor should be differentiated from spindle cell variant of myoepithelioma, while clear cell adenocarcinoma and mucoepidermoid carcinoma should be considered in the differential diagnosis of clear cell variant of myoepithelioma [1, 12]. Simple surgical excision is treatment of choice for benign myoepithelioma. Recurrence is very rare in benign myoepithelioma. Our case did not show any signs of recurrence during 6 months follow up.

## 4. Conclusion

Myoepitheliomas are rare benign tumors most commonly presenting as slowly growing asymptomatic masses. The other salivary glands tumor like pleomorphic adenoma and adenoid cystic carcinoma should be kept in the differential diagnosis of this tumor. Various enhancement patterns of myoepithelioma are seen on contrast enhanced CT, which could be helpful in differentiating it from other soft tissue tumors.
